# Proteomics analysis reveals novel insights into the mechanism of hepatotoxicity induced by *Tripterygium wilfordii* multiglycoside in mice

**DOI:** 10.3389/fphar.2022.1032741

**Published:** 2022-11-17

**Authors:** Yingying Miao, Qin Zhang, Zihang Yuan, Jie Wang, Yunxia Xu, Yuanyuan Chai, Min Du, Qinwei Yu, Luyong Zhang, Zhenzhou Jiang

**Affiliations:** ^1^ New Drug Screening Center, Jiangsu Center for Pharmacodynamics Research and Evaluation, State Key Laboratory of Natural Medicines, China Pharmaceutical University, Nanjing, China; ^2^ Center for Drug Research and Development, Guangdong Pharmaceutical University, Guangzhou, China; ^3^ Key Laboratory of Drug Quality Control and Pharmacovigilance, Ministry of Education, China Pharmaceutical University, Nanjing, China

**Keywords:** *Tripterygium wilfordii* multiglycoside, hepatotoxicity, label-free proteomics, intestinal immune network for IgA production, gut-liver axis

## Abstract

*Tripterygium wilfordii* multiglycoside (GTW), extracted and purified from the peeled roots of *T. wilfordii* Hook.f. (TwHF), is a well-known traditional Chinese medicine and applied to various autoimmune diseases clinically. However, it has been reported to cause severe liver injury. At present, the mechanism underlying GTW-induced hepatotoxicity remain poorly defined. Here, we evaluated the effects of GTW on mouse liver and elucidated the associated mechanisms *via* label-free proteomics combined with bioinformatics analysis. Male C57BL/6J mice were randomly divided into normal group, a low-dose GTW (70 mg/kg) group and a high-dose GTW (140 mg/kg) group. After 1-week administration, GTW dose-dependently induced hepatotoxicity. Further analysis showed that GTW could act on the intestinal immune network for IgA production pathway, which plays an important role in maintaining intestinal homeostasis and influences the crosstalk between gut and liver. Western blots confirmed that GTW could decrease pIgR protein expression in the liver and ileum, and, as a result, the secretion of IgA into gut lumen was reduced. Further validation showed that intestinal barrier integrity was impaired in GTW-treated mice, promoting bacteria transferring to the liver and triggering proinflammatory response. Our study demonstrated that gut-liver axis may play a vital part in the progression of GTW-induced hepatotoxicity, which provides guidance for basic research and clinical application of GTW.

## 1 Introduction

Drug-induced hepatotoxicity is one of the major reasons for liver injury, the most serious of which is acute liver failure characterized by severe hepatocyte death (Andrea Iorga, 2017). Herbal drugs, with complex and diverse constituents, are effect triggers of liver injury leading to acute and chronic liver diseases (Stournaras and Tziomalos, 2015). *Tripterygium wilfordii* multiglycoside (GTW) is a well-known Chinese herbal medicine that is extracted and purified from the peeled roots of *T. wilfordii* Hook.f. (TwHF) and mostly used as tablets in clinical. The clinical dosage of GTW tablets is 1–1.5 mg/kg/day for adults (Gong et al., 2020). According to the standard of GTW tablets (WS3–B-33350-98-2011), stipulated by National Medical Products Administration (China), the contents of triptolide and wilforlide A in each GTW tablet (10 mg) shall not exceed and not less than10 μg, respectively (Dai et al., 2022). Because of its anti-inflammatory and immune-suppressive effects, GTW has been widely applied to various autoimmune diseases, for example, systemic lupus erythematosus (Zhou et al., 2018), nephrotic syndrome (Xu and Jiang, 2009) and rheumatoid arthritis (Tao et al., 2001; Marks, 2011). However, the adverse effects of GTW, especially liver injury, restrict its clinical application (Zhang et al., 2016; Liu et al., 2018). Since the liver is responsible for metabolism and detoxification, it is imperative to explore the mechanisms of GTW-induced liver injury for its safe clinical use.

Although studies have reported that GTW could cause liver damage when administered chronically or at very high doses (Zhang et al., 2012; Peng et al., 2021), generally, investigations on GTW-induced hepatotoxicity and associated molecular mechanisms are still few. Therefore, the effects of GTW on liver and the underlying mechanisms require further investigation.

Proteomics currently emerges as a comprehensive and powerful tool for detecting functional proteins and discovering molecular targets, therefore, it is more and more widely used to explore the pharmacological and toxicological mechanisms of herbal medicine (Bennett and Devarajan, 2018; Zhang et al., 2022). Numerous studies on drug-induced liver injury have been reported to identify differentially expressed proteins (DEPs) and explore associated mechanisms *via* label-free proteomics (Satoh et al., 2014; Dragoi et al., 2018). Although a previous study has used proteomics to detect DEPs in the livers of mice administered triptolide (TP), an essential bioactive but toxic component of TwHF (Li et al., 2017), at present, there are almost no investigations on liver proteome changes in GTW-induced hepatotoxic models.

In the present study, we evaluated the effects of GTW on mice livers and elucidated the associated mechanisms *via* label-free proteomics combined with bioinformatics analysis. By analyzing the identified DEPs and enriched functional pathways between the control and GTW groups, we found that GTW could act on the intestinal immune network for IgA production pathway, suggesting an important role of gut-liver axis in mediating GTW-induced hepatotoxicity. To our knowledge, our research was the first indication of the effects of GTW on mice liver proteome, which is expected to provide more insights into the mechanism of GTW-induced hepatotoxicity.

## 2 Materials and methods

### 2.1 HPLC analysis of *T. wilfordii* multiglycoside

GTW (>98%, Batch No. 1507702) was obtained from Zhejiang DND Drug Factory (Zhejiang, China). The chemical profile of GTW was determined by HPLC. Standard chemicals including triptolide, triptonide, celastrol, and wilforlide A were purchased from National Institute for Food and Drug Control (Beijing, China), and wilforgine and wilforine were obtained from Chengdu Push Bio-technology Co., Ltd. (Sichuan, China). The conditions of HPLC were listed in Table 1.

**TABLE 1:** The instrumental conditions of HPLC.

Catalog	Instrumental conditions
Colum	Agilent Zorbax SB-C18 (4.6 × 100 mm, 3.5 μm)
Mobile phase	Water containing acetonitrile (A)
Flow rate	0.75 ml/min
Elution	0–10 min: 20% A
	10–15 min: from 20% to 30% A
	15–40 min: 30% A
	40–50 min: from 30% to 40% A
	50–60 min: from 40% to 55% A
	60–90 min: from 55% to 85% A
	90–100 min: from 85% to 20% A
	100–110 min: 20% A
Injection volume	10 μl
Analytical wavelength	218 nm

### 2.2 Animals and experimental protocols

Male C57BL/6J mice (6–8 weeks) were supplied by Shanghai SLAC Laboratory Animal Co., Ltd. (Shanghai, China). Mice were housed in conditions with controlled light (12 h light/dark cycle), temperature (24 ± 2°C), and humidity (50%–60%) and had adequate food and tap water. All experiments on mice were performed under the guidelines of Ethical Committee of China Pharmaceutical University (Ethics approval No. 2022-05-003).

GTW in powder was suspended in 0.5% CMC-Na and administered to mice by gavage. Based on the resource equation method (Charan and Kantharia, 2013), one of the methods of sample size calculation in animal studies, 18 male C57BL/6J mice were randomly divided into the control group, GTW-L and GTW-H groups, with 6 mice in each group. The dosage and dosing time of GTW administration were chosen based on our previous studies (Zhang et al., 2012; Miao et al., 2019; Yuan et al., 2019). The doses selected for GTW in animal experiments were 70 mg/kg (GTW-L) and 140 mg/kg (GTW-H), which are approximately 5 times and 10 times mouse equivalent dosage (mouse equivalent dose was 9.1–13.65 mg/kg/day), respectively. After GTW administration for 1 week, mice were sacrificed.

### 2.3 Biochemical and histopathological examinations

Serum ALT, AST, and ALP detection kits were obtained from Whitman Biotech (Nanjing, China). All examinations were performed in accordance with the protocols.

Fragments from mice tissues were fixed in 4% paraformaldehyde overnight, embedded in paraffin, and then sliced for H&E and immunohistochemistry (IHC) staining to observe the pathological changes.

### 2.4 Immunofluorescence

Mouse tissue sections were fixed in 4% paraformaldehyde for 15 min, washed in PBS and permeabilized with 0.1% Triton X-100 for 10 min. After blocked with 5% goat serum for 1 h at room temperature, they were incubated with primary antibodies at 4°C overnight. Then, samples were incubated with secondary antibodies and DAPI (1:1000) at room temperature for 1 h, and further imaged with a confocal laser scanning microscope (Olympus, Lake Success, LY). The following antibodies were used: mouse anti-occludin (1:200, #33-1500, Thermo Fisher), APC anti-mouse F4/80 antibody (1:50, #123115, BioLegend), Alexa Fluor 488-conjugated anti-mouse IgG (1:1000, ab150113, abcam).

### 2.5 Label-free quantitative proteomics and bioinformatics analysis

Label-free proteomics analysis of liver samples from the control and GTW-H (140 mg/kg) groups was performed with the help of Novogene Co., Ltd. (Beijing, China). The experimental procedures and methods were listed in Supplementary Materials and Methods.

Gene Ontology (GO) annotation analysis of the identified DEPs was performed using Database for Annotation, Visualization and Integrated Discovery (DAVID) (version 6.8) (Huang et al., 2009). Kyoto Encyclopedia of Genes and Genomes (KEGG) enrichment analysis of the DEPs was performed using KEGG Orthology-Based Annotation System (KOBAS) (version 3.0) (Bu et al., 2021), and the connection between pathways were visualized using Cytoscape with ClueGO and CluePedia applications (Bindea et al., 2009; Mlecnik and Bindea, 2013). *p*-value less than 0.05 was considered statistically significant.

The Search Tool for the Retrieval of Interacting Genes (STRING) database (http://www.string-db.org/) was used to establish protein-protein interaction (PPI) network of the DEPs (Szklarczyk et al., 2017). After visualization of the network by Cytoscape, the densely connected protein modules in the PPI network was detected using Molecular Complex Detection (MCODE) app with default parameter settings (Bader and Hogue, 2003). Furthermore, bioinformatics analysis for the top cluster, including biological process annotation and KEGG enrichment pathway analysis, was conducted according to the above-mentioned methods.

### 2.6 Western blot analysis

Mouse tissue samples were homogenized in RIPA buffer (Beyotime, China) supplemented with protease and phosphatase inhibitors (Bimake, China). After protein quantification assayed with a BCA method (Beyotime, China), the lysis was mixed with loading buffer (Bio-Rad, CA, United States) and denatured by heat. Then, protein was separated on SDS–PAGE and subsequently transferred onto PVDF membranes by electroblotting. Next, the membrane was blocked with 5% bovine serum albumin for 1 h at room temperature, followed by incubating with primary antibody at 4°C overnight. Finally, the membrane was incubated with HRP-conjugated polyclonal secondary antibody at room temperature for 1 h, and further visualized using an ECL detection kit (Tanon, Shanghai, China). The antibodies used in the study are as follows: rabbit anti-β-ACTIN (AC026, ABclonal), rabbit anti-pIgR (A6130, ABclonal), mouse anti-Occludin (#33-1500, Thermo Fisher).

### 2.7 Real-time quantitative PCR

Mouse tissue samples were homogenized in Trizol reagent for RNA extraction. Approximately 1 μg of RNA was converted to cDNA after quantification with Nanodrop 2000 (Thermo, DE, United States). Target genes were analyzed by real-time PCR (RT-PCR) using SYBR Green on Stepone Plus (Thermo, DE, United States) with specific primers. Primers used were listed on Table 2, and the β-actin gene was used for normalization.

**TABLE 2:** Primer sequences used for real-time PCR.

Gene		Primer sequence (5′ to 3′)
*16S rRNA*	Forward	AGA​GTT​TGA​TCC​TGG​CTC​AG
	Reverse	TGC​TGC​CTC​CCG​TAG​GAG​T
*Tlr2*	Forward	ACA​GCA​AGG​TCT​TCC​TGG​TTC​C
	Reverse	GCT​CCC​TTA​CAG​GCT​GAG​TTC​T
*Tlr4*	Forward	AGC​TTC​TCC​AAT​TTT​TCA​GAA​CTT​C
	Reverse	TGA​GAG​GTG​GTG​TAA​GCC​ATG​C
*Tnfα*	Forward	GGT​GCC​TAT​GTC​TCA​GCC​TCT​T
	Reverse	GCC​ATA​GAA​CTG​ATG​AGA​GGG​AG
*β-actin*	Forward	CAT​TGC​TGA​CAG​GAT​GCA​GAA​GG
	Reverse	TGC​TGG​AAG​GTG​GAC​AGT​GAG​G

### 2.8 Enzyme-linked Immunosorbent assay (ELISA)

#### 2.8.1 Detection of IgA in feces and serum

Mouse fecal samples were suspended in PBS containing protease inhibitor, and centrifuged at 12,000 × g for 15 min. After centrifugation, the supernatant was collected and diluted 1/1000 for fecal IgA ELISA. Serum was diluted 1/2000 for IgA ELISA. Mouse IgA ELISA kit was obtained from Neobioscience Technology Co., Ltd. (Shenzhen, China).

#### 2.8.2 Serum TNFα detection

Mouse serum TNFα concentration was measured using commercially available ELISA kits according to the manufacturer’s protocols. Mouse TNFα ELISA kit was obtained from Neobioscience Technology Co., Ltd. (Shenzhen, China).

### 2.9 Fluorescence *in situ* hybridization detection of bacteria in liver

Fluorescence *in situ* hybridization (FISH) analysis was conducted according to the protocol from GenePharma (Shanghai, China). Briefly, liver frozen sections were fixed in 4% paraformaldehyde for 15 min, washed in PBS, and incubated with proteinase K for 15 min. Then, slices were washed and incubated with 5 ng/μl of probes at 37°C for 12–16 h. After incubation, slices were washed in PBS and then stained with DAPI at room temperature for 20 min, and further imaged with a confocal laser scanning microscope (Olympus, Lake Success, LY). A mix of probes was synthesized by GenePharma, as shown in Table 3. The non-Eub probe was used as a negative control.

**TABLE 3:** FISH probes.

Probe	Fluorochrome	Sequence (5′ to 3′)
EUB338 Ⅰ	FAM	GCTGCCTCCCGTAGGAGT
EUB338 Ⅱ	FAM	GCAGCCACCCGTAGGTGT
EUB338 Ⅲ	FAM	GCTGCCACCCGTAGGTGT
Non-EUB	CY3	ACTCCTACGGGAGGCAGC

### 2.10 Statistical analysis

Data are presented as mean ± SEM. The differences between two groups were analyzed using Student’s *t*-test, and the differences among multiple groups were analyzed using one-way analysis of variance (ANOVA). Statistical analysis and graphing were performed using GraphPad Prism 9 software (GraphPad Software, Inc., San Diego, CA, United States). *p*-value less than 0.05 was considered statistically significant.

## 3 Results

### 3.1 Fingerprint analysis of *T. wilfordii* multiglycoside by HPLC

**FIGURE 1:**
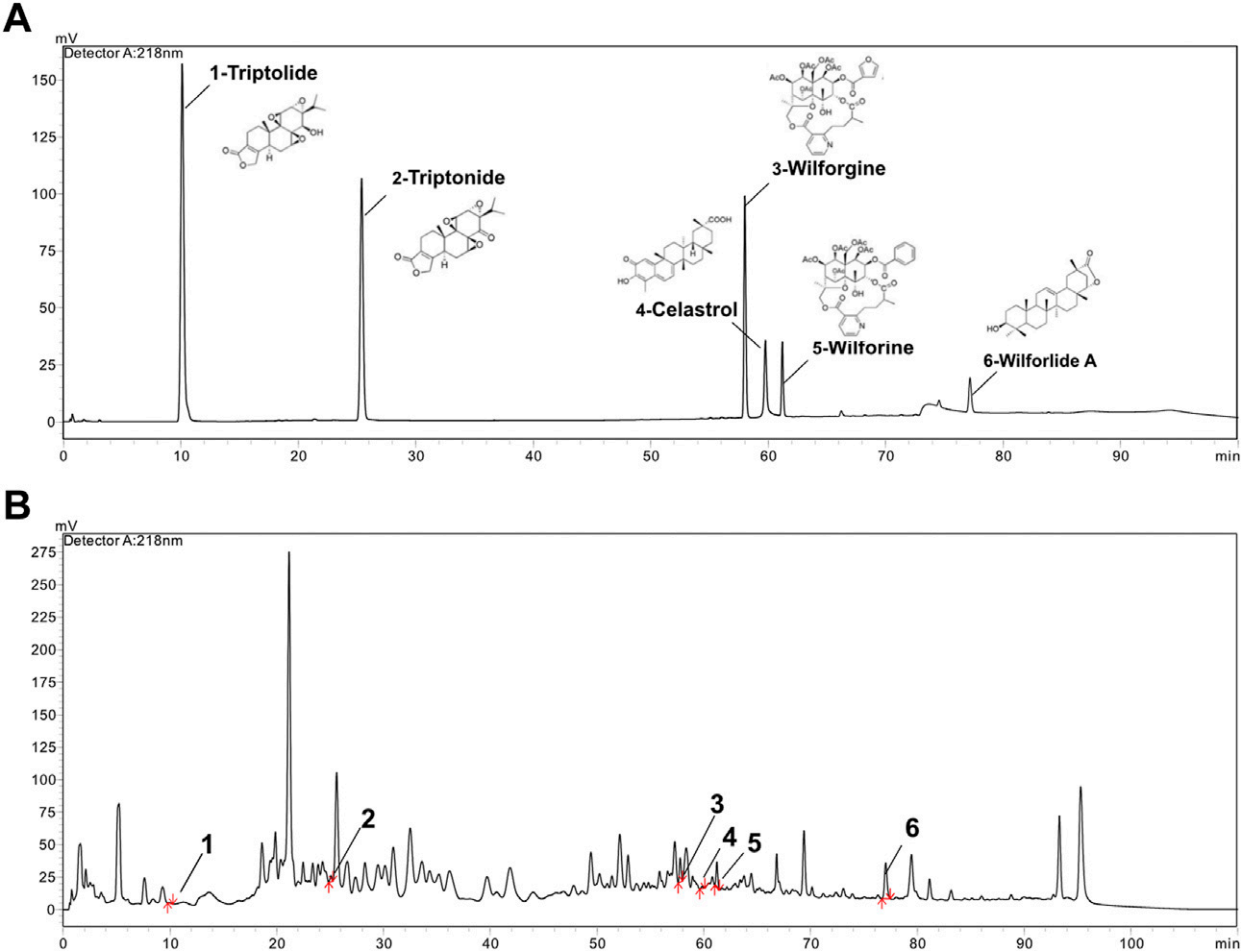
HPLC analysis of standard mixture and GTW. **(A)** The chromatograph of triptolide, triptonide, wilforgine, celastrol, wilforine, and wilforlide A standard mixture. **(B)** The HPLC chromatograph of GTW.

In this study, the fingerprint of GTW was determined by an HPLC method, and components including triptolide, triptonide, wilforgine, celastrol, wilforine, and wilforlide A were detected and quantified (Figure 1). According to Table 4, the contents of triptolide and wilforlide A meted the quality standards of GTW (WS3-B-3350-98-2011).

**TABLE 4:** Content assay of six components in GTW.

Name	Content (μg/g)	Content (‰)	Ct (min)	Standard (‰)
Triptolide	5.25	0.005	10	≤1
Triptonide	83.75	0.084	26	—
Wilforgine	479.75	0.480	57.8	—
Celastrol	67	0.067	60.1	—
Wilforine	1870.75	1.871	60.9	—
Wilforlide A	4460.75	4.461	77.4	≥1

### 3.2 *T. wilfordii* multiglycoside dose-dependently caused hepatotoxicity in mice

**FIGURE 2:**
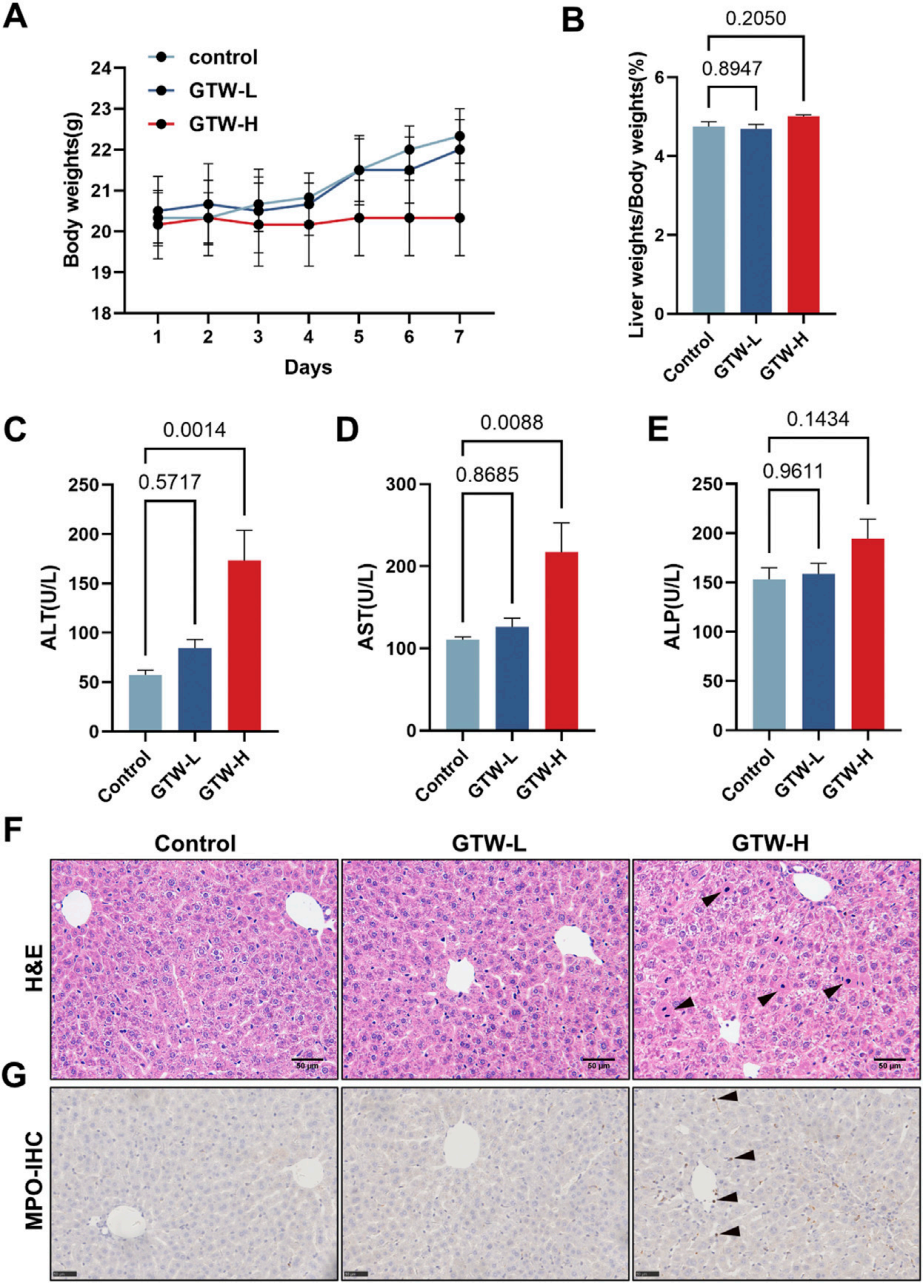
GTW administration caused liver injury in mice. **(A)** Changes in body weights (*n* = 6). **(B)** Liver tissue index calculated by tissue and body weights (*n* = 6). **(C–E)** Serum aminotransferase levels, including ALT, AST, and ALP (*n* = 6). **(F)** Representative H&E staining images of liver sections (scale bar = 50 μm), and nuclear pyknosis was pointed with black arrows. **(G)** Representative immunohistochemical measurements of liver in each group (scale bar = 50 μm). MPO positive areas were pointed with black arrows. Data are presented as mean ± SEM. *p* < 0.05 was considered statistically significant.

The body weights of experimental mice were recorded during 1-week administration of GTW. Compared with the control, GTW-H could prevent mice weight gain (Figure 2A). Meanwhile, liver tissue index of 140 mg/kg GTW-treated mice was slightly increased (Figure 2B). Serum aminotransferase levels were measured to evaluate whether GTW caused liver injury. As shown in Figures 2C–E, only 140 mg/kg GTW caused a significant increase in serum ALT and AST levels, suggesting hepatocellular injury. The effects of GTW on liver structure were examined by histopathological evaluation. Compared with the normal group, nuclear pyknosis was observed in the liver sections of GTW-H group (Figure 2F), suggesting apoptotic cell death. In addition, increased MPO immunopositive areas revealed recruitment and infiltration of neutrophils in GTW-H treated liver (Figure 2G). Taken together, these results demonstrate that GTW administration could induce hepatotoxicity in a dose-dependent manner.

### 3.3 Label-free quantification of mouse liver proteins after *T. wilfordii* multiglycoside administration

**FIGURE 3:**
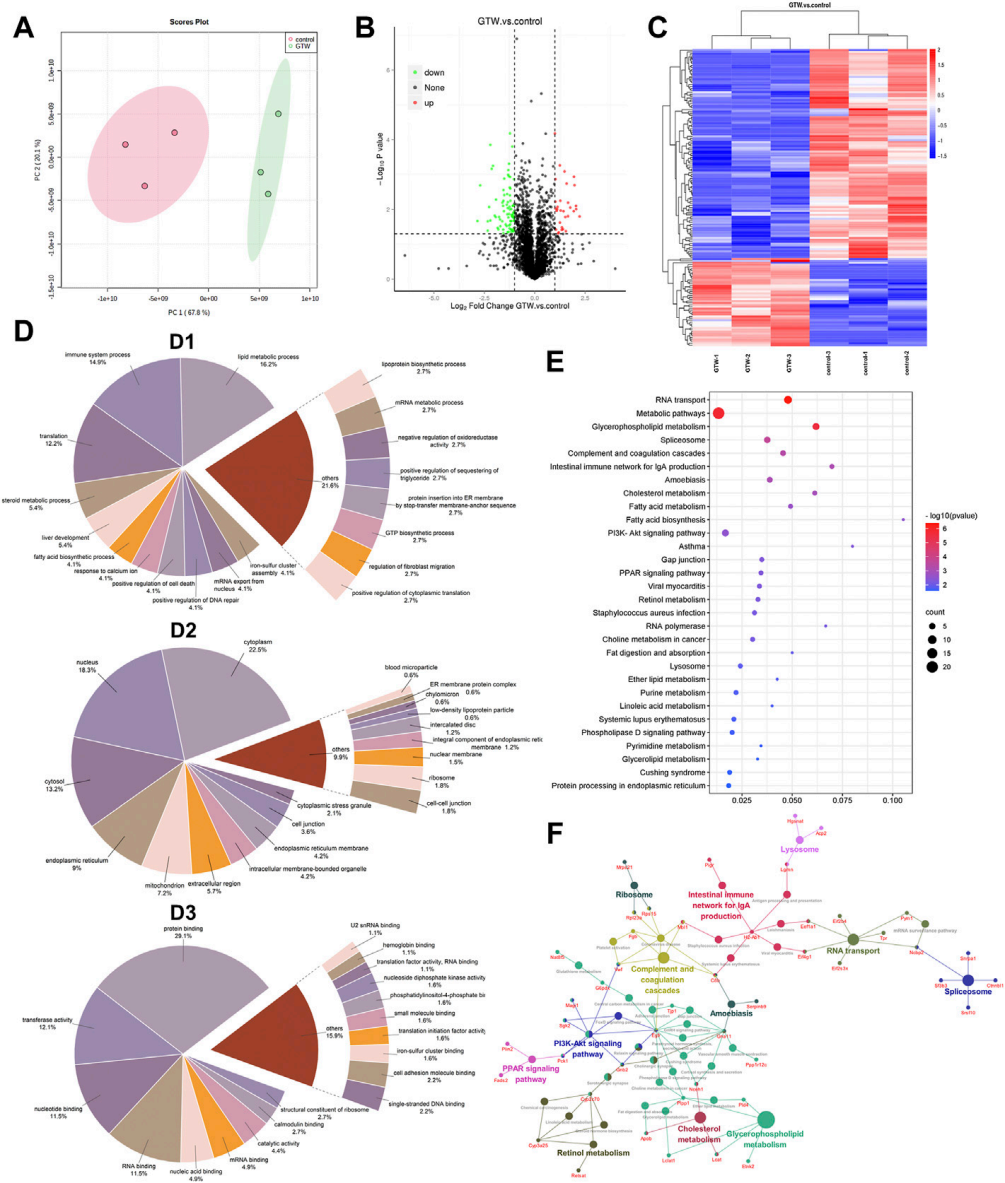
Results of proteomics combined with bioinformatics analysis of mice livers after GTW exposure. **(A)** PCA analysis of identified proteins. **(B)** Volcano plot. Proteins upregulated or downregulated were presented as red or green points, respectively, and black points represented proteins with no significant changes (|log2FC| ≥ 1 and *p* < 0.05). **(C)** Heatmaps of DEPs. Rows represented DEPs and columns represented samples with three biological replicates. The signal values of DEPs were shown as colored bars, that is, the high expressed DEPs were colored in red, and the low expressed proteins were colored in blue. **(D)** GO annotation classification analysis of DEPs. (D1: Biological process. D2: Cellular component. D3: Molecular function.) **(E)** KEGG enrichment analysis of DEPs (*p* < 0.05). The ordinate represented the pathway term, and the abscissa represented the ratio of DEP numbers involved in a KEGG pathway to all identified protein numbers annotated in this pathway. The number of DEPs was represented by dot size, and the *p*-value was indicated by dot color. **(F)** The connection between pathways.

To obtain a comprehensive overview of the mechanism underlying GTW-induced hepatotoxicity, label-free quantitative proteomics was performed to identify the DEPs between the control group and GTW-H (hereinafter referred to as GTW) group. After three replicated biological analyses with high reliability, 3,670 proteins were recognized, of which 2,641 proteins were identified in both groups (Supplementary Table S1). Meanwhile, the proteomics profile of GTW group showed a clear separation from the control group (Figure 3A). Next, we screened DEPs by comparing protein abundance between the groups (fold change (FC) ≥ 2 and ≤ 0.5; *p*-value < 0.05) and identified 155 DEPs with 46 upregulated and 109 downregulated proteins (Figure 3B; Supplementary Table S2). The accuracy of the selected DEPs was measured by cluster analysis. As shown in Figure 3C, the identified DEPs were clearly distinguished between the two groups, suggesting that these DEPs may represent key changes in mice livers after GTW administration.

### 3.4 Bioinformatics analysis of differentially expressed proteins

#### 3.4.1 Gene ontology annotation analysis

The GO annotation analysis on DEPs was carried out and classified into three GO terms: biological process (BP), cellular component (CC), and molecular function (MF). According to BP results (Figure 3D1), lipid metabolic process (16.2%), immune system process (14.9%), and translation (12.2%) were the top three processes. In CC analysis (Figure 3D2), the largest proportions of DEPs were assigned to the cytoplasm (22.5%), nucleus (18.3%), and cytosol (13.2%). In MF analysis (Figure 3D3), protein binding (29.1%), transferase activity (12.1%), nucleotide binding (11.5%), and RNA binding (11.5%) accounted for the major proportions.

#### 3.4.2 Kyoto encyclopedia of genes and genomes pathway enrichment analysis

To better understand the functional categories, KEGG pathway analysis of DEPs was conducted. The top 30 pathways were shown in Figure 3E, including RNA transport, glycerophospholipid metabolism, spliceosome, complement and coagulation cascade, and intestinal immune network for IgA production. Figure 3F shows the connection between them.

#### 3.4.3 Protein-protein interaction analysis and significant cluster identification

**FIGURE 4:**
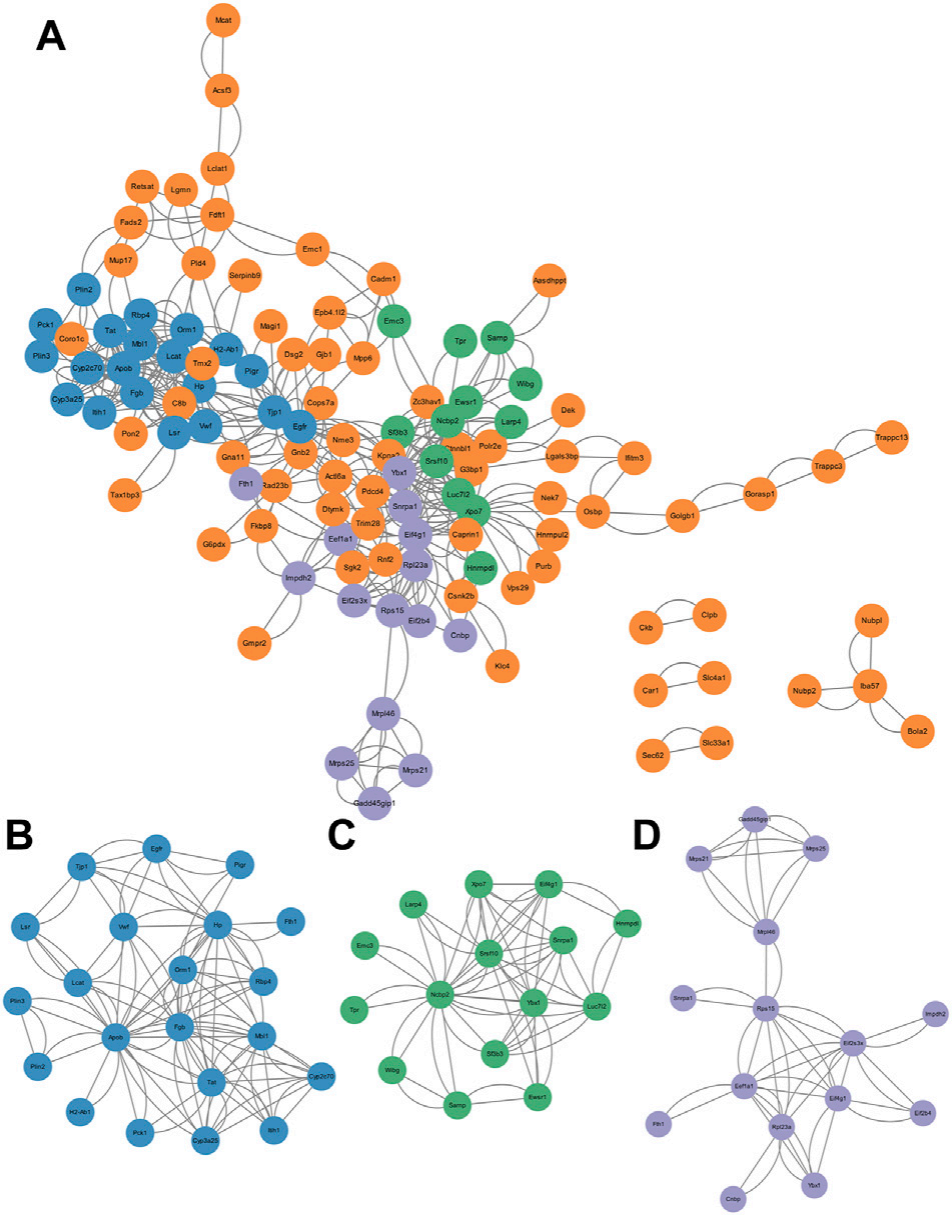
Construction and cluster analysis of PPI network. **(A)** PPI network of DEPs visualized by Cytoscape. **(B–D)** The top three clusters screened by MCODE, including cluster 1 (score = 5.4, nodes = 21, edges = 108), cluster 2 (score = 4.4, nodes = 15, edges = 62), and cluster 3 (score = 3.6 nodes = 15, edges = 50).

To further determine the key pathways involved in GTW-induced hepatotoxicity, PPI analysis and significant cluster identification were performed. By using the STRING database, PPI network of the DEPs was conducted to explore their interactions. We observed that a total of 114 nodes and 432 edges showed interconnectivity (Figure 4A). The significant interconnected regions in this PPI network were analyzed, and the top three clusters were recognized (Figures 4B–D; Supplementary Table S3). Cluster 1 (Figure 4B) had the highest score, indicating that it would play an important role in regulating this PPI network, thus, BP annotation and KEGG pathway analysis were performed. According to Table 5, cluster 1 was closely related to immune system process, response to lipopolysaccharide, and response to bacterium, suggesting that abnormal immune response would be the main reason for GTW-induced hepatotoxicity. Further KEGG analysis showed that intestinal immune network for IgA production pathway would be involved in the liver toxicity.

**TABLE 5:** GO_BP annotation and KEGG pathway analysis of nodes in cluster 1.

Category	Term	Count	*p*-value
GOTERM_BP	Liver development	3	0.005
GOTERM_BP	Steroid metabolic process	3	0.007
GOTERM_BP	Immune system process	4	0.014
GOTERM_BP	Response to lipopolysaccharide	3	0.015
GOTERM_BP	Response to bacterium	3	0.033
KEGG_pathway	Complement and coagulation cascades	3	0.000
KEGG_pathway	Intestinal immune network for IgA production	2	0.000
KEGG_pathway	Cholesterol metabolism	2	0.000
KEGG_pathway	Linoleic acid metabolism	2	0.000
KEGG_pathway	Adherens junction	2	0.000
KEGG_pathway	PI3K-AKT signaling pathway	3	0.000

IgA, transported by polymeric immunoglobulin receptor (pIgR), is a major immunoglobulin isotype in the gut and plays a crucial role in maintaining gut barrier (Gutzeit et al., 2014). The interaction between gut and liver makes liver vulnerable to disrupted intestinal homeostasis (Seki and Schnabl, 2012). Intestinal barrier dysfunction could promote bacterial translocation to the liver and trigger proinflammatory response, contributing to the development of liver diseases (Wang et al., 2015; Tripathi et al., 2018). Thus, we assumed that GTW should affect the intestinal immune network for IgA production pathway and disturb intestinal homeostasis, contributing to abnormal immune response in livers of GTW-treated mice.

### 3.5 *T. wilfordii* multiglycoside acted on the intestinal immune network for IgA production pathway

**FIGURE 5:**
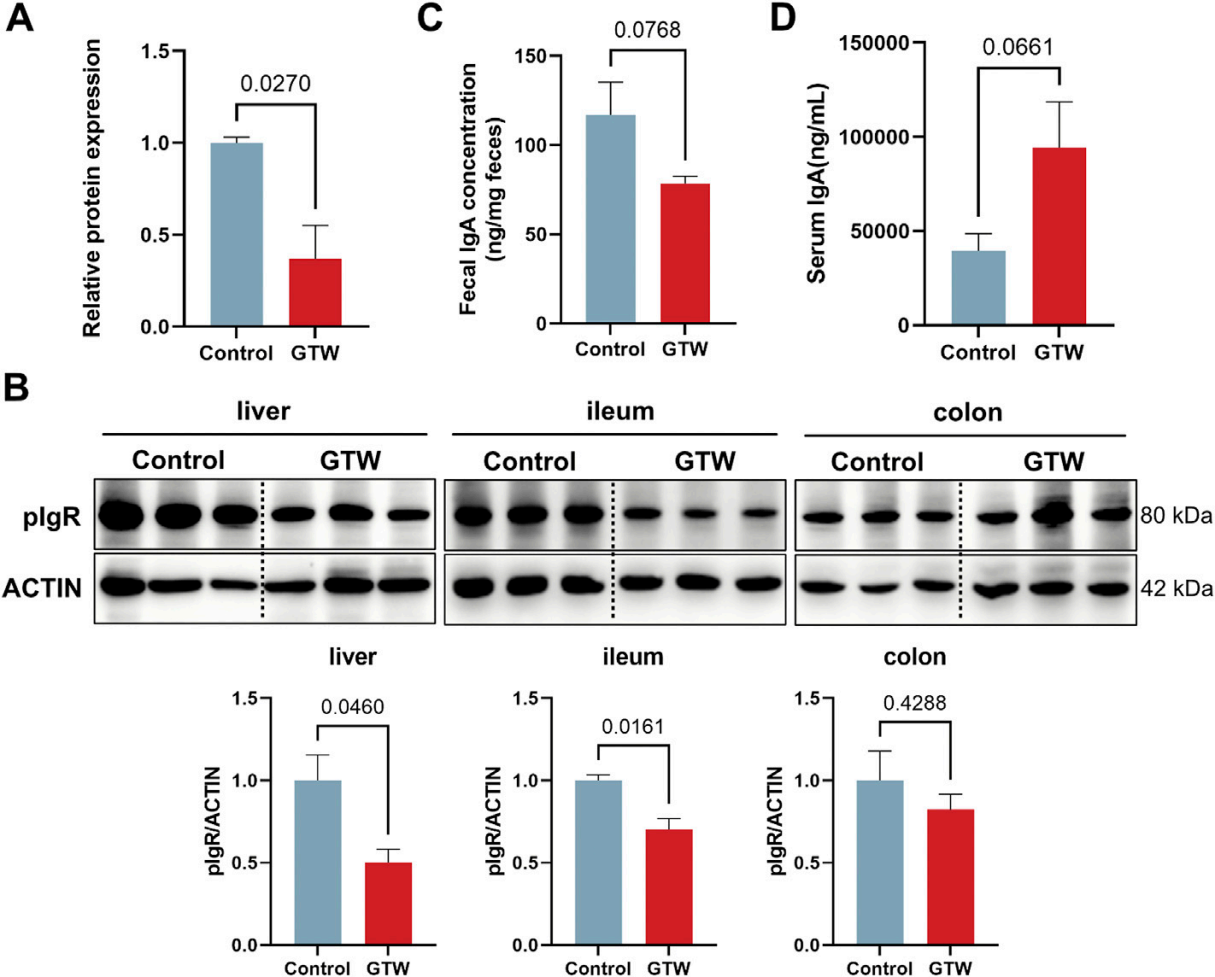
GTW affected the activation of intestinal immune network for IgA production pathway. **(A)** Relative pIgR protein expression according to proteomics results (*n* = 3). **(B)** The protein levels of pIgR in liver, ileum and colon were determined by western blot (*n* = 3). **(C)** IgA levels in feces (*n* = 5) and **(D)** serum (*n* = 5) were measured after GTW treatment. The band density was calculated using ImageJ software. Data are presented as mean ± SEM. *p* < 0.05 was considered statistically significant.

Next, we evaluate the effects of GTW on the intestinal immune network for IgA production pathway. Results from proteomics indicated that GTW could downregulate pIgR protein expression (Figure 5A). Immunoblots confirmed that pIgR protein in liver was decreased in GTW group (Figure 5B). In addition, we assessed the expression of pIgR in gut and found that pIgR protein expression was reduced in the ileum but not significantly changed in the colon after GTW exposure (Figure 5B). As a consequence, the reduction of pIgR resulted in a decreased secretion of IgA into the gut lumen (Figure 5C), and a buildup of serum IgA compared to the normal group (Figure 5D). These results suggest that GTW could inhibit intestinal immune network for IgA production pathway *via* disturbing pIgR/IgA system.

### 3.6 *T. wilfordii* multiglycoside-treated mice displayed intestinal barrier impairment

**FIGURE 6:**
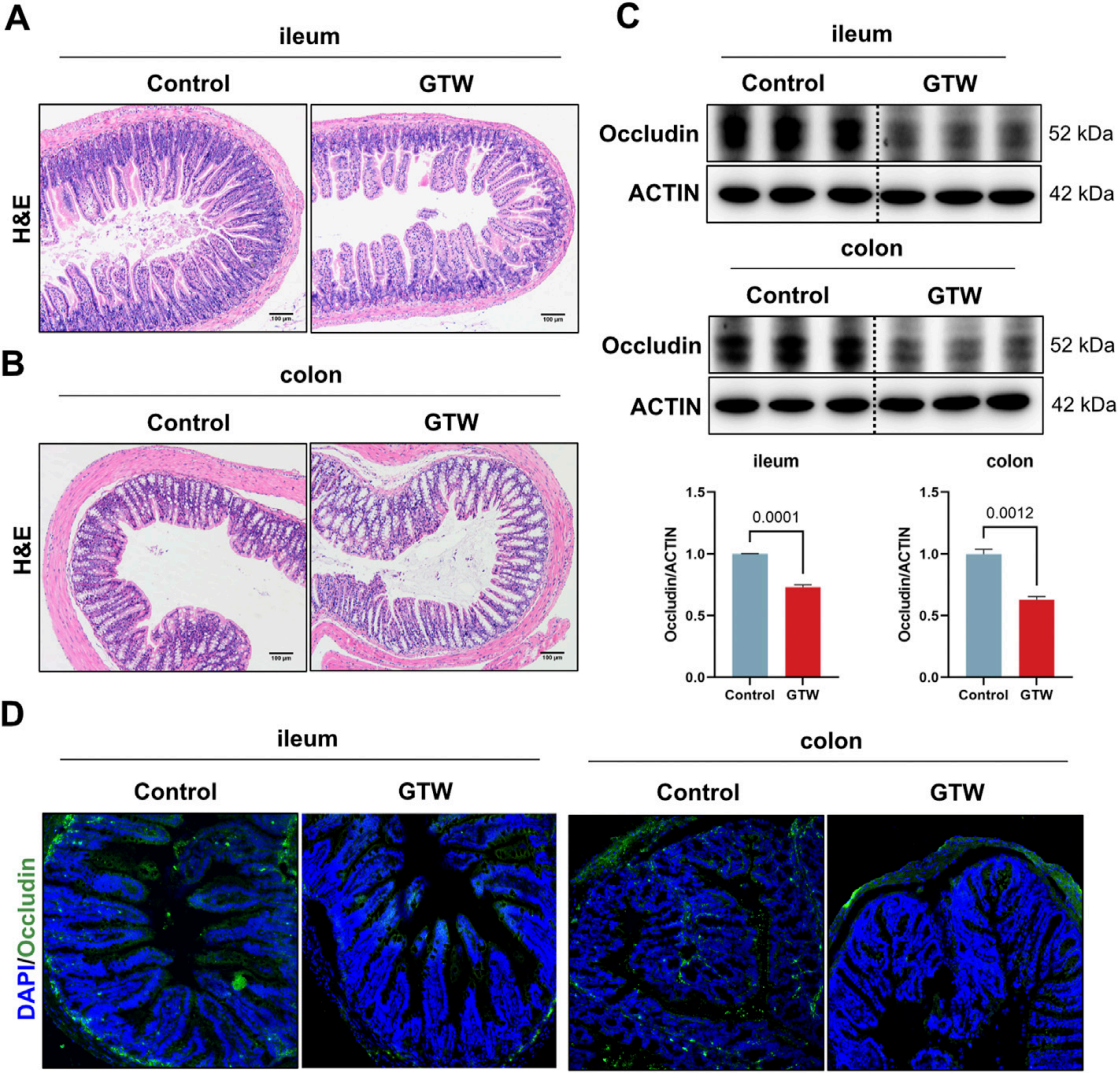
The effects of GTW on intestinal barrier integrity. **(A,B)** Representative H&E staining images of ileum and colon tissues (scale bar = 100 μm). **(C)** The protein levels of occludin in the ileum and colon were determined by western blot (*n* = 3). **(D)** Representative images of immunofluorescent staining of occludin (green) in ileum and colon sections (20x). The nuclei were visualized with DAPI (blue). The band density was calculated using ImageJ software. Data are presented as mean ± SEM. *p* < 0.05 was considered statistically significant.

The intestinal immune network for IgA production pathway plays a crucial role in maintaining mucosal homeostasis and gut barrier (Brown and Kloppel, 1989; Mantis et al., 2011). Therefore, we assessed whether the intestinal barrier was altered in GTW-treated mice. Histopathological examination showed that in GTW-treated mice ileum, the villi were sparse and broken and the crypt depth was reduced (Figure 6A). In addition, GTW could damage the colon, manifested by extensive loss of epithelial cells, destruction of crypts, and inflammatory cell infiltration (Figure 6B). As a reliable sign of intestinal barrier disruption, we further analyzed the expression of tight junction protein occludin in the ileum and colon. As shown in Figure 6C, the ileal and colonic protein levels of occludin were decreased. Consistently, the fluorescence intensity of intestinal epithelial occludin was much lower in GTW-treated mice than in control mice (Figure 6D). These results point towards impaired intestinal barrier integrity and show consistency with disrupted pIgR/IgA system.

### 3.7 Intestinal barrier impairment contributed to *T. wilfordii* multiglycoside-induced hepatotoxicity

**FIGURE 7:**
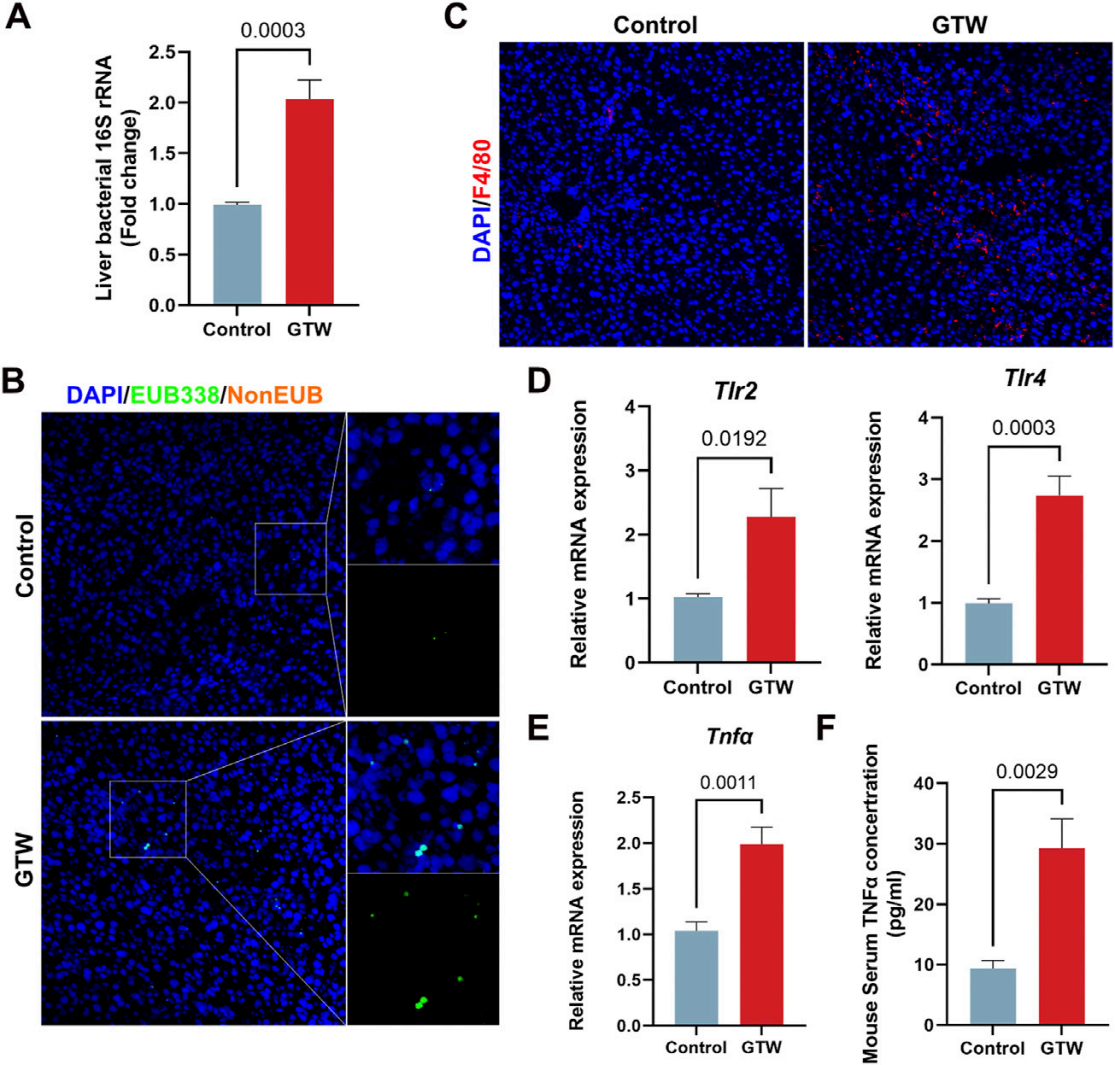
GTW administration promoted bacterial translocation to the liver and triggered hepatic inflammation. **(A)** Liver bacterial 16S rRNA expression measured by RT-PCR (*n* = 6). **(B)** Bacteria detected in the liver using FISH with probes EUB338 targeting eubacteria 16S rRNA region (20x). The nuclei were stained with DAPI (blue) and bacteria stained with EUB338 (green). **(C)** Representative images of immunofluorescent staining of F4/80 (red) in liver (20x). DAPI was used to visualize nuclei (blue). **(D)** Hepatic TLR2 and TLR4 mRNA expression levels were detected by RT-PCR (*n* = 6). **(E)** The expression levels of proinflammatory cytokine TNFα in liver were measured using RT-PCR analysis (*n* = 6). **(F)** Serum TNFα concentration in GTW-treated mice were detected by ELISA (*n* = 6). Data are presented as mean ± SEM. *p* < 0.05 was considered statistically significant.

The liver, a key immune organ, is positioned to receive gut-derived products *via* the portal vein, implying that it could be severely affected by a disrupted intestinal homeostasis (Schnabl, 2013; Wang et al., 2021). The disruption of gut barrier allows bacterial translocation (Albillos et al., 2020). We thus detected bacteria in the liver. The 16S rRNA gene expression in GTW-treated mice livers were significantly increased (Figure 7A), in addition, FISH analysis confirmed that bacterial invasion to the liver was promoted after GTW exposure (Figure 7B). In agreement with this result, clear signs of inflammatory response in GTW-treated mice livers were observed, with an increase of F4/80 labeled macrophages (Figure 7C) and of hepatic TLR2 and TLR4 mRNA levels (Figure 7D). In addition, increased mRNA levels of proinflammatory cytokine TNFα (Figure 7E) and increased serum TNFα concentration (Figure 7F) further confirmed liver inflammation. Thus, these findings suggest that GTW-caused intestinal barrier impairment could increase bacterial translocation to the liver and trigger hepatic inflammation, which may contribute to GTW-induced liver toxicity.

## 4 Discussion

GTW is a stable extract purified from the peeled roots of TwHF and extensively applied for autoimmune diseases in China. Three major ingredients, including diterpenoids (e.g., triptolide and triptonide), sesquiterpene alkaloids (e.g., wilforgine and wilforine), and triterpenoids (e.g., celastrol and wilforlide A), are thought to be contributed to the pharmacodynamics activities of GTW (Luo et al., 2016). In the study, we used an HPLC method to characterize the fingerprint of GTW, and calculated the contents of 6 representative components. It was found that among them, wilforlide A showed the highest content (the mean value was 4460.75 μg/g) while triptolide showed the lowest (the mean value was 5.25 μg/g). These results meted the quality standards of GTW (WS3-B-3350-98-2011) and showed consistency with previous studies (Luo et al., 2016).

The clinical application of GTW is restricted due to its adverse effects, especially liver injury. TP is concerned as an essential bioactive but toxic component of TwHF (Li et al., 2014b). Previous studies demonstrated that liver damage caused by TP was mainly associated with mitochondrial dysfunction (Fu et al., 2011), oxidative stress (Li et al., 2014a), abnormal lipid and glucose metabolism (Wang et al., 2013; Jiang et al., 2016), and inflammation (Wang et al., 2014). Compared to TP, studies about GTW-induced liver injury are few and associated signaling mechanisms remain poorly understood. In addition, to study the toxic effects of GTW, it is more appropriate to take GTW as a whole than to focus only on TP. In this study, we conducted experiments in male C57BL/6J mice to evaluate GTW-induced hepatotoxicity. ALT and AST are two reliable markers reflecting hepatocellular injury while ALP reflects the extent of cholestasis (Cockeram, 1998). After 1-week administration, GTW at high dose significantly increased serum ALT and AST levels but had minor effects on ALP, indicating hepatocellular damage. Further examinations confirmed that GTW could cause hepatocyte apoptosis and liver inflammation. Similar to our results, a recent study reported that GTW induced hepatotoxicity in zebrafish *via* increased inflammation and enhanced apoptosis (DUAN et al., 2021). In fact, we also investigated the hepatotoxic effects of GTW on female mice. By detecting serum aminotransferase levels, we found that whereas there was no significant gender difference, the hepatotoxic effects of GTW showed a better dose-dependent manner in male mice than in female (Supplementary Figure S1).

Based on label-free proteomics combined with bioinformatics analysis, intestinal immune network for IgA production pathway was predicted to be involved in GTW-induced liver toxicity. IgA is a major immunoglobulin isotype in the gut and plays a crucial role in maintaining intestinal homeostasis (Mantis et al., 2013). Transport of IgA from the lamina propria to the mucosal surface and the release of secretory IgA onto the gut lumen require pIgR, a protein expressed on mucosal epithelial cells (Kaetzel et al., 1991; Schneeman et al., 2005). Besides lamina propria-produced IgA, liver-derived IgA is also an important source of total gut IgA in mouse, which is secreted to bile by pIgR on hepatocytes and cholangiocytes (Brown and Kloppel, 1989; Turula and Wobus, 2018). Secretory IgA has been reported to favor the maintenance of gut barrier by regulating microbiota composition, preventing bacteria invasion, and downregulating immune responses in the intestinal mucosal (Strugnell and Wijburg, 2010; Mantis et al., 2011). Deficiency in secretory IgA would alter gut microbiome and increase intestinal permeability (Inamine and Schnabl, 2018). Accumulating evidence indicates that controlling gut microbial composition is critical for maintaining intestinal homeostasis (Albillos et al., 2020). Gut bacterial dysbiosis can lead to a direct interaction between the microbiota and epithelial cells, promoting inflammatory response and enhancing gut permeability, thereby increasing bacterial translocation and affecting the development of chronic liver diseases, including ALD, NAFLD and NASH(Mao et al., 2015; Cheng et al., 2018; Wang et al., 2020; Wang et al., 2021). Results from our study demonstrate that GTW could significantly reduce pIgR protein expression in the liver and ileum, as a result, a reduction in fecal IgA was observed. In agreement with reduced fecal IgA levels, serum IgA concentration was increased, which was consistent with previous study (Shimada et al., 1999). These findings suggest that GTW could act on intestinal immune network for IgA production pathway *via* inhibiting pIgR/IgA system, it raises the possibility that GTW would disrupt intestinal homeostasis, which might be associated with altered microbiota composition, and as a consequence, the increased intestinal inflammation, the impaired gut barrier integrity and the enhancement of intestinal permeability.

We thus assess whether GTW caused intestinal barrier dysfunction. Histopathological analysis showed that GTW administration caused damage to ileum and colon, in addition, obvious inflammation was observed in the colon of GTW-treated mice. Tight junctions, sealing adjacent intestinal epithelial cells together, play a central role in gut barrier maintenance (Chelakkot et al., 2018; Lee et al., 2018). Numerous studies have reported that damage to tight junctions contributes to intestinal barrier dysfunction (Lee, 2015; Chen et al., 2017). In our study, we found that the expression of tight junction protein occludin in the ileum and colon were decreased after GTW exposure, indicating impaired intestinal barrier integrity. The liver, the first organ for receiving gut-derived products through the portal vein, is vulnerable to disrupted intestinal homeostasis (Inamine and Schnabl, 2018). Gut barrier disruption enhances intestinal permeability and promotes bacteria and/or bacterial products (e.g., lipopolysaccharides) transferring to the liver, triggering a proinflammatory cascade, thereby inducing or exacerbating a range of hepatic diseases (Albillos et al., 2020; Ghosh et al., 2020). In our study, increased bacteria were found in the livers of GTW-treated mice while livers of control mice were harboring few bacteria. Toll-like receptors (TLR) have been shown to recognize microbial pattern recognition receptors and stimulate immune response (Kawasaki and Kawai, 2014), we thus assessed hepatic TLR2 and TLR4 expression. In agreement with increased bacterial translocation, TLR2 and TLR4 mRNA levels in GTW-treated mice livers were significantly elevated. In addition, F4/80 immunofluorescent staining revealed more macrophages in the livers of GTW-treated mice, and MPO immunohistochemical staining revealed increased recruitment and infiltration of neutrophils. The proinflammatory response was further confirmed by the increase of hepatic TNFα mRNA expression and of serum TNFα concentration. Taken together, our study suggests that GTW could disrupt intestinal barrier integrity, promoting the translocation of bacteria to the liver and increasing hepatic inflammation. Furthermore, our findings suggest a key role of gut-liver axis in GTW-induced liver injury. In fact, according to clinical reports, GTW has the highest incidence of gastrointestinal upset, ranging from diarrhea, nausea, vomiting, and even colitis (Zhang et al., 2016), it raises the possibility that GTW could disrupt gut barrier and thereby affect the progression of liver toxicity. However, the effects of GTW on gut microbiome and the role of intestinal microbiota dysbiosis in GTW-induced hepatotoxicity require validation in our further study, in addition, our study just did a preliminary exploration, more experiments need to be conducted to investigate the crosstalk between gut and liver in GTW-induced hepatotoxic model and to identify potential molecular targets.

Outcomes obtained from this study reveal novel insights into the mechanism of GTW-induced hepatotoxicity, but, inevitably, there are limitations. First, these findings were based on the analysis of livers in mice, which may have inconsistencies with clinical results. Second, the effects of GTW on gut microbiome and the crosstalk between gut and liver injury required further exploration. Third, in addition to the gut-liver axis, other functional pathways and targets related to GTW-induced hepatotoxicity need to be validated. Furthermore, potential ingredients and/or toxic core structures, contributing to hepatotoxicity, need to be explored to promote the clinical application of GTW.

## 5 Conclusion

Our findings demonstrate that GTW could induce liver injury. Based on label-free proteomics combined with bioinformatics analysis, we found that GTW may act on intestinal immune network for IgA production pathway and impair intestinal barrier integrity, therefore, the bacterial translocation to the liver and hepatic inflammation were increased. In summary, our study reveals a novel insight into the mechanism of GTW-induced hepatotoxicity that the crosstalk between gut and liver may play a crucial part in the progression of GTW-induced liver toxicity. However, more experimental data are needed to verify this.

## Data Availability

The datasets of proteomics analysis presented in this study can be found in ProteomeXchange Consortium with the dataset identifier PXD038084.
