# Supplementary material for: Proteomics analysis reveals novel insights into the mechanism of hepatotoxicity induced by Tripterygium wilfordii multiglycoside in mice
Source: Front Pharmacol. 2022 Nov 17;13:1032741. doi: 10.3389/fphar.2022.1032741 (PMC9712739; doi:10.3389/fphar.2022.1032741)
Supplement: Supplementary file 1 [file DataSheet3.docx]

The original data was uploaded in jianguoyun.

Please refer to the link blow:

https://www.jianguoyun.com/p/DTWz62oQiO7xChjw790EIAA
