# Supplementary material for: Proteomics analysis reveals novel insights into the mechanism of hepatotoxicity induced by Tripterygium wilfordii multiglycoside in mice
Source: Front Pharmacol. 2022 Nov 17;13:1032741. doi: 10.3389/fphar.2022.1032741 (PMC9712739; doi:10.3389/fphar.2022.1032741)
Supplement: Supplementary file 2 [file DataSheet1.docx]

Supplementary Material

# Supplementary Materials and methods

## Label-free quantitative proteomic analysis

### Protein extraction

Liver sample was ground individually in liquid nitrogen and lysed with PASP lysis buffer (100 mM NH_4_HCO_3_, 8 M Urea, pH 8) followed by ultrasonication for 5 min on ice. The lysate was centrifuged at 12000 g for 15 min at 4℃ and the supernatant was reduced with 10 mM DTT for 1 h at 56℃, and subsequently alkylated with sufficient IAM for 1 h at room temperature in the dark. Then samples were completely mixed with 4 times volume of precooled acetone and incubated at -20℃ for at least 2 h. Samples were then centrifuged at 12000 g for 15 min at 4℃ and the precipitation was collected. After washing with 1mL cold acetone, the pellet was dissolved by dissolution buffer (8 M Urea, 100 mM TEAB, pH 8.5).

### Trypsin digestion

Each protein sample was added with DB lysis buffer (8 M Urea, 100 mM TEAB, pH 8.5) to make up the volume to 100 μL, then trypsin and 100 mM TEAB buffer were added, sample was mixed and digested at 37 °C for 4 h. Then trypsin and CaCl_2_ were added and digested overnight. Formic acid was mixed with the digested sample to adjust the pH < 3. Then sample was centrifuged at 12000 g for 5 min at room temperature. The supernatant was slowly loaded to the C18 desalting column and washed with washing buffer (0.1% formic acid, 3% acetonitrile) 3 times, then added with elution buffer (0.1% formic acid, 70% acetonitrile). The eluents of each sample were collected and lyophilized.

### LC-MS/MS analysis

The lyophilized powder was dissolved in 10 μL solution A (water containing 0.1% formic acid) and centrifuged at 14000 g for 20 min at 4 ℃, then separated by an Evosep One UHPLC system. The separated peptides were analyzed by Q Exactive^TM^ HF-X mass spectrometer (Thermo, USA) with Nanospray Flex™ (ESI) ion source, spray voltage at 2.1 kV, and ion transport capillary temperature at 320°C. Full scan range from m/z 350 to 1500 with a resolution of 60000 (at m/z 200), an automatic gain control (AGC) target value was 3×106 and a maximum ion injection time was 20 ms. The top 40 highest abundant precursors in the full scan were selected and fragmented by higher energy collisional dissociation (HCD) and analyzed in MS/MS, where resolution was 15000 (at m/z 200), the automatic gain control (AGC) target value was 1×10^5^, the maximum ion injection time was 45 ms, normalized collision energy was set as 27%, intensity threshold was 2.2×10^4^, and the dynamic exclusion parameter was 20 s.

### Protein identification and quantitation

The proteomics data generated by mass spectrometry in RAW file format were searched and identified. The all resulting spectra were searched against Uniprot database (<http://www>.uniprot.org) using Proteome Discoverer 2.2 (Thermo, USA). The search parameters are set as follows: mass tolerance for precursor ion was 10 ppm and mass tolerance for product ion was 0.02 Da. Carbamidomethyl was specified as fixed modifications, oxidation of methionine was specified as dynamic modification, and acetylation was specified as N-terminal modification in Proteome Discoverer 2.2. A maximum of 2 missed cleavage sites were allowed.

In order to improve the quality of analysis results, the software Proteome Discoverer 2.2 further filtered the retrieval results: peptide spectrum matches (PSMs) with a credibility of more than 99% were identified PSMs. The identified protein contains at least 1 unique peptide. The identified PSMs and protein were retained and performed with FDR no more than 1.0%. The protein quantitation results were statistically analyzed by *t*-test. The proteins whose quantitation significantly different between GTW and control groups, (p < 0.05 and |log_2_FC| > 1), were defined as differentially expressed proteins (DEPs).

# Supplementary Figures


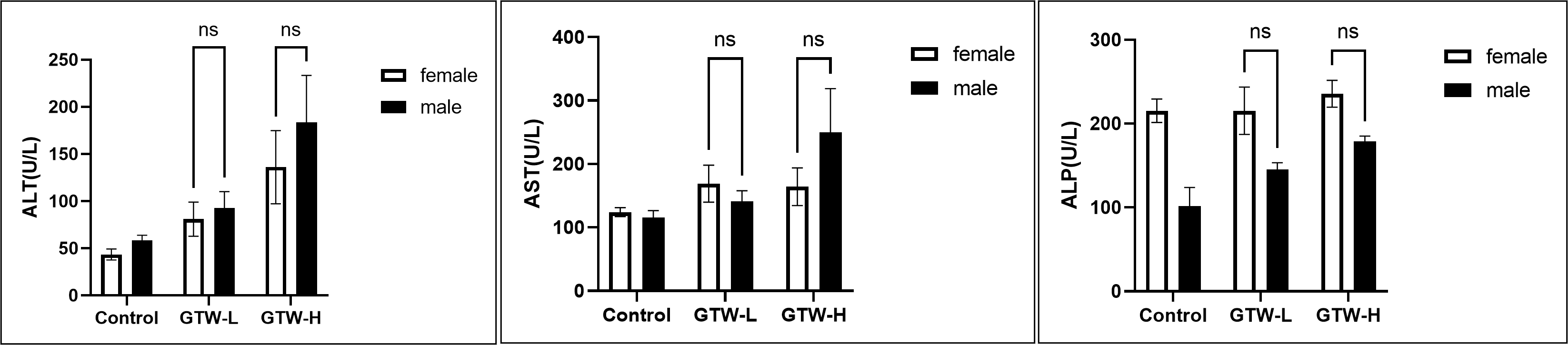


**Figure S1.** Serum aminotransferase levels, including ALT, AST, and ALP were measured after GTW administration in female and male mice (n = 3). Data are presented as mean ± SEM. *P* < 0.05 was considered statistically significant.
